# Effects of Ageing on the Eye Structure and Function 2019

**DOI:** 10.1155/2020/5192491

**Published:** 2020-03-19

**Authors:** Alejandro Cerviño, Jose F. Alfonso, Hema Radhakrishnan, Jose M. González-Meijome, Rune Brautaset

**Affiliations:** ^1^Department of Optics and Optometry and Vision Sciences, University of Valencia, Valencia, Spain; ^2^Fernández-Vega Ophthalmological Institute, Oviedo, Spain; ^3^University of Manchester, Manchester, UK; ^4^Department of Physics (Optometry), University of Minho, Braga, Portugal; ^5^Karolinska Institutet, Stockholm, Sweden

The world's population is growing older, with persons over the age of 65 being the fastest-growing age group. According to data from World Population Prospects 2019 [1], one in 11 people in the world was over the age of 65 in 2019 (9%), and this number is expected to increase to one in six (16%) by 2050 since life expectancy is also expected to increase from current 72.6 years to 77.1. In 2018, for the first time in history, persons aged 65 or above outnumbered children under five years of age.

Aging is a natural process that affects the function of many organs, including the eyes, having both structural and functional consequences for the visual system, affecting all ocular structures and causing a variety of effects. Improving quality of life as well as reducing age-related disability is of increasing importance for health systems. Determining the mechanisms behind age-related conditions and healthy ageing may help creating tools to improve early detection of involutive changes and prognosis, delay the onset of disease, and allow the optimization of resources through the treatment at earlier stages of the condition.

The eye is usually considered a window for findings of systemic disease, due to its high metabolic demands to keep transparency on its media, its sensitivity to vascular compromise, and the possibility of observing the different structures in a noninvasive manner. When age-related changes turn into age-related conditions, they manifest into eye disease, such as age-related macular degeneration (AMD), cataract, or pseudoexfoliation (PEX) syndrome. Several signalling pathways have been implicated in the aging process. In the paper titled “Repressed Wnt Signaling Accelerates the Aging Process in Mouse Eyes,” Y. Zhang et al. suggest that disruption of Wnt signalling homeostasis in the eye is associated with accelerated aging.

One of the important aspects to understand is the anatomical change in the different ocular structures occurring with healthy aging and how they differ from age-related disease. In the paper titled “Panoramic Observation of Crystalline Lenses with 25 MHz Ultrasonography,” W. Xue and H. Zou use 25 MHz B-scan ultrasound to assess change in the entire lens contour and the radii of curvatures of the central anterior and posterior lens surfaces. They confirm that the lens grows equatorially and axially with age while its central anterior lens surface steepens and its posterior central surface curvature does not change.

Age-related overproduction or overaggregation of elastic microfibrillar components on the lens results in PEX. The role of its inheritance has been explored in the past in different populations, and several genes were found likely to play a role in PEX, such as clusterin. In the paper titled “CLU Polymorphisms in Patients with Pseudoexfoliation Syndrome in Polish Population,” H. Lesiewska et al. investigate this possible association in the Polish population concluding that clusterin variants may contribute to the risk of PEX.

Various retinal degenerative processes, such as AMD, concur with selective outer retinal degeneration. In the paper titled “Outer Retinal Layers' Thickness Changes in Relation to Age and Choroidal Thickness in Normal Eyes,” M. K. Abdellatif et al. identify and correlate age-related changes in outer retinal layers' thickness and choroidal thickness in normal eyes using spectral-domain optical coherence tomography and investigate factors affecting these changes. They report significant thinning of retinal pigment epithelium/outer-segment layer thickness with increasing age.

Similarly, in the paper titled “Distribution of Choroidal Thinning in High Myopia, Diabetes Mellitus, and Aging: A Swept-Source OCT Study,” F. A. Bartol-Puyal et al. analyse the distribution of choroidal thinning in high myopia, diabetes mellitus, and aging, showing different thinning pattern that may help identifying conditions. They report the choroidal thickness pattern in young healthy individuals as resembling a mountain range; with age, a mountain peak; in high myopia, an inverted gorge; and in aged type 2 diabetic patients, gathered hills. The thicker the zone is in healthy subjects, the thinner it becomes with any pathology.

Age-related anatomical changes as described have functional consequences. Age-related changes in the eye may arise from several sources and affect visual function in different ways. Difficulty in adaptation to light level changes is a common complaint associated with ageing, particularly regarding night driving, and with significant impact in quality of life. Photostress is one of the methods that test adaption of the visual system and might be, therefore, useful to determine functional differences due to impairment of photopigment regeneration. In the paper titled “An Alternative Psychophysical Diagnostic Indicator of the Aging Eye,” J. D. Rodriguez et al. explore the use of photostress with the addition of flickering stimuli, less dependent on refractive error or straylight, for assessing the health of the aging retina. They report that photostress recovery of flicker sensitivity under mesopic conditions agrees with subject-reported complaints in reduced luminance conditions after exposure to bright lights, such as night driving, highlighting the potential usefulness of the method for the clinical assessment in diseased eyes.

Finally, older persons generally have greater susceptibility to infections than younger adults. Aging is associated with immune dysfunction, especially in cell-mediated immunity, and elderly persons also suffer from a variety of chronic disorders, some of which affect the integrity of host resistance to infections.

Elderly individuals have an increased susceptibility to skin infections due to age-related anatomical, physiological, and environmental factors. The skin of the elderly is structurally and functionally different from that of other age groups. Mites are found on almost all normal adult skin. Demodex is a host-specific obligate parasite, and clinical observations based on large samples are important for exploring the relationship between its presence and clinical signs. In the paper titled “The Prevalence of *Demodex folliculorum* and *Demodex brevis* in Cylindrical Dandruff Patients,” J. Zhong et al. carry out a large sample study in China designed to determine the prevalence of Demodex and the effect of host-related factors such as gender, age, and eyelid inflammation on this prevalence. They report higher prevalence in Demodex spp. in older subjects and greater prevalence on subjects with cylindrical dandruff than healthy subjects. Furthermore, in subjects with cylindrical dandruff, the number of *Demodex spp*. was reported as positively correlated with age and exacerbated the severity of eyelid congestion, providing, therefore, a good clinical reference.

We know that significant challenges remain in understanding the full extent of structural and functional changes in the eye which results from ageing. Some of these changes are outlined in this special issue. We hope that these stimulate ideas which will further advance our understanding of the ageing eye.

*Alejandro Cerviño* *Jose F. Alfonso* *Hema Radhakrishnan* *Jose M. González-Meijome* *Rune Brautaset*
